# Gender-specific mediators of the association between parental education and adiposity among adolescents: the HEIA study

**DOI:** 10.1038/s41598-019-43604-w

**Published:** 2019-05-13

**Authors:** Mekdes K. Gebremariam, Onyebuchi A. Arah, Ingunn H. Bergh, Lene F. Andersen, Yngvar Ommundsen, Torunn H. Totland, Mona Bjelland, May Grydeland, Nanna Lien

**Affiliations:** 1 Department of Epidemiology, Fielding School of Public Health, University of California, Los Angeles (UCLA), Los Angeles, California United States; 2 Department of Nutrition, Faculty of Medicine, University of Oslo, Oslo, Norway; 3 UCLA Center for Health Policy Research, Los Angeles, California United States; 4 California Center for Population Research, UCLA, Los Angeles, California United States; 5 Department of Child Development, Norwegian Institute of Public Health, Oslo, Norway; 6 Department of Coaching and Psychology, Norwegian School of Sport Sciences, Oslo, Norway; 7 Norwegian National Advisory Unit on Ageing and Health, Vestfold Hospital Trust, Tønsberg, Norway; 8 Department of Geriatric Medicine, Oslo University Hospital, Oslo, Norway; 9 Department of Physical Performance, Norwegian School of Sport Sciences, Oslo, Norway

**Keywords:** Epidemiology, Risk factors

## Abstract

Identifying the mechanisms behind socioeconomic inequalities in adiposity among youth is vital for efforts aimed at combating these inequalities. The study explored whether a broad range of behavioral and familial factors mediated the associations between parental education and indicators of adiposity among adolescents. Baseline data from a school-based intervention study conducted in 2007 among 11-year-old adolescents were used. Anthropometric outcomes, physical activity and sedentary time among adolescents were objectively measured. Other behavioral variables and parental waist circumference were self-reported. Mediation analyses were conducted. Among boys, maternal waist circumference (WC), paternal WC and TV viewing mediated 16%, 11.5% and 13% of the association between parental education and adolescent WC. The respective proportions when body fat percentage was used as the outcome variable were 22.5%, 16% and 21%. Among girls, maternal and paternal WC mediated 20% and 14% of the association between parental education and WC. The respective proportions when body fat percentage was used as the outcome variable were 14% and 10%. Other included variables did not play any mediating role. Parental WC was found to be a mediator of socioeconomic differences in adiposity in both genders; underlying mechanisms were however not investigated. Among boys, reducing TV time could contribute to the reduction of social inequalities in adiposity.

## Introduction

In 2016, it was estimated that 50 million girls and 74 million boys worldwide were obese^1^. The prevalence of overweight (OW)/obesity (OB) among Norwegian youth has similarly been found to be high, with around one in five children being OW or obese^2–5^. Some positive trends (i.e. stabilization and even decrease) in the prevalence of childhood OW and OB have been recently documented^6^. However, there is a need for more public health efforts to reverse the pattern of the OW/OB epidemics. Despite these positive trends, a widening of inequalities in OW and OB was found in around half of the studies included in a recent review^6^. Such findings are troubling as childhood OW/OB has several well-documented adverse impacts on health^7–11^; and can be moderately maintained into adulthood^12^. Inequalities in body weight in childhood can thus lead to or exacerbate inequalities in health in childhood as well as adulthood.

Body weight has multiple levels of influence ranging from individual to broader environmental factors, which often interact^13^. The most proximal determinants of body weight include energy balance-related behaviors as well as genetic predisposition. If these factors vary by socioeconomic position, they can potentially lead to (or mediate) socioeconomic inequalities in body weight. A mediator represents an intervening variable in the causal pathway between exposure and outcome^14^. The selection of potential mediators should thus be informed by their previously documented associations with socioeconomic position (exposure) and body weight (outcome). In this regard, sedentary time (ST) has been found to be positively related to OW and OB^15,16^, although associations have not always been consistent^16^. Inverse associations between physical activity (PA) and OW/OB among youth have been found^17,18^. Associations between different dietary behaviors and OW/OB have also been previously documented^19–22^. Socioeconomic differences in these energy balance-related behaviors have also been found in different contexts^23–25^. Parental adiposity is an important correlate of child adiposity, reflecting the role of genetic factors as well the shared obesogenic environment^26–28^. Children from a low socioeconomic background are more likely to have parents who are OW/OB than their counterparts with a higher socioeconomic position^6,29,30^.

Despite the well documented socioeconomic inequalities in body weight among youth^6,29,30^, in-depth knowledge about the specific factors contributing to these inequalities is however limited. The existing literature is marked by shortcomings including the limited use of formal tests of mediation and paucity of data needed to make firm conclusions (e.g. inclusion of a limited number of potential mediators)^31^.

Body mass index (BMI) has been the measure of adiposity most commonly used in studies assessing mediators of socioeconomic differences in adiposity among youth^31^. Existing research suggests that BMI might underestimate socioeconomic differences in childhood obesity compared to other indicators of adiposity^32^. Other measures such as waist circumference (WC) are found to be more important markers of cardio-metabolic disease risks^33,34^. However, WC and BMI might be clinically independent markers of cardio-metabolic risk^35^. Finally, only few previous studies focusing on mediators of socioeconomic inequalities in adiposity have assessed potential gender-related differences^31^.

In summary, there is a scarcity of studies including a broad range of mediators, using rigorous analytical methods, including measures of adiposity other than BMI and conducting gender-specific analyses. The present study aimed to address these gaps. The aims of the study were to explore whether selected dietary behaviors, objectively measured physical activity and sedentary time, screen-based sedentary behaviors and parental adiposity mediated the associations between parental education and measures of adiposity (WC and body fat percentage) among adolescents. Gender-specific analyses were conducted to explore potential differences between genders.

## Methods

### Design and sample

Baseline data from a school-based intervention study, the HEalth In Adolescents (HEIA) study were used^36^.

All schools from the third to fourth largest towns/municipalities in the seven counties surrounding the county of Oslo were invited to participate in the study if they met the study selection criteria (i.e. minimum of 40 enrolled 6th graders). A total of 177 schools were invited and 37 accepted the invitation. All 6^th^ graders (n = 2165) in these 37 schools and their parents/legal guardians were invited to participate in the baseline study in September 2007. Informed consent from parents was obtained for 1589 of the adolescents and 1537 adolescents filled in the baseline questionnaire. Mothers and fathers were also invited to participate in the study; 1260 mothers and 1068 fathers participated.

Informed consent for participation was obtained from school administrators and from the parents of participating children. Adolescents provided assent. The research was carried out in accordance with relevant ethical guidelines and regulations. Ethical clearance was obtained from the Regional Committees for Medical Research in Norway and the Norwegian Social Science Data Service who approved the experimental protocol for the study.

### Data collection and measures

The adolescents answered an internet-based questionnaire, assisted as necessary by research assistants. Anthropometric measurements were conducted by research group members. Parents filled in a paper questionnaire sent home via the adolescents.

### Measures

#### Outcome variables: child anthropometrics

Adolescents’ WC was measured to the nearest 0.1 cm. The measuring tape was positioned midway between the lower rib and the iliac crest at the end of a normal expiration. Tanita scales (using bioelectrical impedance analysis) were used to measure body fat percentage (Tanita TBF-300, Tanita Corporation of America, Illinois, USA). The adolescents were measured in light clothing and barefoot.

#### Exposure variable: parental education

Parental education was gathered as part of the parental informed consent for the adolescent. Educational status of the parent with the longest education or else the one available was used in the analyses. Parental education was initially categorized into three: low (equal to or less than 12 years), medium (university/college (up to 4 years)) and high (university/college (more than 4 years)). However, there were no significant differences between the latter two categories in terms of the associations explored. Thus, these two categories were merged into a “high education” category.

#### Mediators

The mediators included in this study were: sedentary behaviors (TV/DVD use, computer/electronic game use), dietary behaviors (consumption of fruits, vegetables, unhealthy snacks and sugar-sweetened soft drinks), objectively measured PA and ST, maternal WC and paternal WC.

Four questions with pre-coded answer categories were asked to assess usual TV/DVD and computer/electronic game use: “How many hours do you usually watch TV and/or DVD on a normal weekday?” The same question was asked for a normal weekend day. The six answer categories ranged from half hour per day to five or more hours per day. The two questions on computer/electronic game use were formulated in the same way as for TV/DVD, but the six answer categories ranged from no use to four or more hours per day. Weekly scores for TV/DVD and computer/electronic games were calculated by summing hours reported for an average weekday (multiplied by five) and an average weekend day (multiplied by two).

Consumption of fruit and vegetables (raw and cooked) was assessed by three frequency questions with eight categories ranging from never/seldom to three times per day or more. The questions assessing the intake of fruits and vegetables were validated among 11-year-olds with a 7-day food record as the reference method. They were found to have a satisfactory ability to rank subjects according to their intake of fruits and vegetables^37^. Frequency of consumption of unhealthy snacks (candies, chocolate, sweet biscuits, buns/muffins and salty snacks (e.g. potato chips)) was assessed using questions with seven categories ranging from never/seldom to two times per day or more. Intake of sugar-sweetened soft drinks (sum of sugar-sweetened carbonated soft drinks and fruit drinks) during weekdays was assessed using two frequency questions with categories ranging from never/seldom to every weekday. Amount was assessed in glass (from one to four or more, with one glass = 1.67 deciliters). The adolescents were also asked the amount of sugar-sweetened soft drinks consumed in the weekends using two questions with categories ranging from never/seldom to 7 glasses or more. Weekday and weekend values were summed up to create a weekly intake variable. The questions assessing the intake of sugar-sweetened soft drinks have been validated among 9- and 13-year-olds using a 4-day pre-coded food diary as the reference method. Moderate correlation coefficients were obtained^38^.

Adequate test-retest correlation coefficients were obtained for the questions assessing dietary intake and screen-based sedentary behaviors in a separate test-retest study conducted 10–14 days apart among 111 6^th^ graders prior to the main data collection^36^.

Objectively measured ST and PA were assessed using accelerometers (ActiGraph GT1M/model 7164, LLC, Pensacola, FL, USA). The adolescents were instructed to wear accelerometers for 5 consecutive days, during all waking hours, except when doing water activities (as monitors were not waterproof). ST was defined as activity at intensities less than 100 counts per minute (cpm), which equals the intensity of sitting or lying down (<1.5 MET). Moderate to vigorous physical activity (MVPA) was defined as all activity at intensities above 2000 cpm. This threshold is approximately equivalent to a walking pace of 4 km/h in youth. These cut-offs have been used in previous studies^39,40^. ST and MVPA were expressed as min/day of accelerometer activity measured. In addition, in order to account for variation in accelerometer wear time, these variables were divided by wear time of the accelerometer (min/day), yielding variables for percent time spent sedentary and percent time spent on MVPA. Details of the instructions provided to students and of the data handling and extraction procedures are available elsewhere^41^.

Parental WC was reported by parents. A flexible measuring tape was enclosed with the questionnaires sent to parents. Written guidelines (including an illustration) on how to do the measurement were also included.

#### Potential confounders

Family structure was divided into two categories: those living with two parents versus those with other living arrangements. Participants were divided into either ethnic Norwegian or ethnic minority. Ethnic minorities were defined as those having both parents born in a country other than Norway^42^.

The Pubertal Development Scale (PDS), based on the pubertal category scores defined by Carskadon and Acebo^43^, was used to assess pubertal status. The students self-reported this information on a separate paper questionnaire. PDS for boys included body hair growth, voice, and facial hair. For girls, PDS included body hair growth, breast development, and menarche. The categories were: pre-pubertal, early pubertal, mid-pubertal, late pubertal or post-pubertal. For the analyses, dichotomization into pre/early pubertal and mid/late/post-pubertal was made for girls and into pre-pubertal and early/mid/late/post-pubertal for boys. Such categorization was suggested by a study that compared PDS and the Sexual Maturation Scale^44^.

### Statistical analyses

The study aims were first explored using descriptive statistics. Parental educational differences in anthropometric outcomes, potential mediators and potential confounders included were assessed using independent samples t-test for continuous variables and chi-squared test for categorical variables (Table 1). The associations between the potential mediators and the outcome variables (WC and body fat percentage) were explored using regression analyses (Table 2).

**Table 1:** Characteristics of study participants stratified by gender and parental education (N = 1537)*.

	Girls			Boys		
	Means (SD) or %		p value*	Means (SD) or %		p value^#^
	High education	Low education		High education	Low education	
Ethnicity (% Norwegian)	95	92	0.09	96	91	0.006
Family structure (% living with 2 parents)	83	71	0.001	82	70	0.001
WC (cm)	61.8 (6.0)	63.4 (7.0)	0.002	63.5 (6.2)	65.5 (8.0)	<0.001
Body fat %	18.7 (7.2)	20.7 (7.8)	0.001	14.6 (5.3)	16.2 (7.0)	0.001
TV viewing (hrs/wk)	10.8 (6.6)	11.7 (7.4)	0.11	11.7 (6.6)	12.9 (7.9)	0.03
Computer use (hrs/wk)	6.9 (5.5)	7.5 (6.1)	0.20	9.3 (6.3)	10.4 (7.4)	0.06
SSB intake (dl/wk)	8.1 (8.4)	11.8 (11.9)	<0.001	10.8 (11.3)	15.0 (14.4)	<0.001
Fruit intake (t/wk)	10.8 (6.9)	10.2 (7.2)	0.34	9.2 (6.6)	9.4 (7.2)	0.76
Vegetable intake (t/wk)	11.8 (9.1)	11.5 (9.7)	0.67	10.3 (8.8)	11.1 (10.7)	0.28
Unhealthy snacks (t/wk)	4.1 (3.6)	5.0 (5.4)	0.01	4.6 (4.2)	5.8 (6.4)	0.002
% time spent sedentary	63.6 (6.1)	62.4 (6.3)	0.06	61.9 (7.0)	61.2 (6.0)	0.26
Daily sedentary time (min)	494.2 (63.5)	490.3 (71.9)	0.52	487.4 (67.6)	483.0 (70.9)	0.49
% time spent on MVPA	7.8 (2.4)	7.7 (2.4)	0.66	9.43 (3.01)	9.5 (3.6)	0.79
Daily MVPA time (min)	60.6 (18.6)	59.9 (18.0)	0.59	74.1 (24.4)	75.0 (28.3)	0.70
Maternal WC (cm)	80.8 (10.1)	83.2 (11.8)	0.02	79.4 (9.6)	83.5 (12.1)	<0.001
Paternal WC (cm)	92.7 (9.4)	94.8 (10.3)	0.04	92.9 (9.1)	95.1 (10.4)	0.03

^*^n varies between variables due to missing data, in particular for the measures of physical activity, sedentary time, maternal WC, paternal WC.

^#^P values for differences between parental educational groups (low vs high).

Values are presented as means and standard deviations or as %.

MVPA: moderate to vigorous physical activity; SSB: sugar-sweetened beverages; WC: waist circumference.

**Table 2:** Association between behavioral and familial correlates and waist circumference and body fat % among study participants (N = 1537)*.

	Boys				Girls			
	Waist circumference		Body fat%		Waist circumference		Body fat%	
	B (CI)	p value	B (CI)	p value	B (CI)	p value	B (CI)	p value
TV viewing (hrs/wk)	0.22 (0.15, 0.29)	<0.001	0.20 (0.14, 0.26)	<0.001	0.05 (−0.02, 0.12)	0.12	0.07 (−0.01, 0.16)	0.08
Computer use (hrs/wk)	0.16 (0.09, 0.24)	<0.001	0.15 (0.09, 0.21)	<0.001	0.07 (−0.01, 0.15)	0.07	0.06 (−0.03, 0.16)	0.21
SSB intake (dl/wk)	0.02 (−0.01, 0.07)	0.22	0.03 (−0.07, 0.06)	0.11	0.004 (−0.04, 0.05)	0.86	−0.005 (−0.06, 0.05)	0.86
Fruit intake (times/wk)	−0.04 (−0.11, 0.03)	0.29	−0.03 (−0.09, 0.03)	0.35	0.02 (−0.05, 0.09)	0.58	−0.02 (−0.10, 0.06)	0.63
Vegetable intake (t/wk)	−0.05 (−0.10, 0.001)	0.056	−0.02 (−0.06, 0.03)	0.49	0.006 (−0.04, 0.06)	0.82	0.02 (−0.08, 0.04)	0.53
Unhealthy snacks (t/wk)	−0.02 (−0.13, 0.08)	0.65	−0.05 (−0.14, 0.04)	0.24	−0.07 (−0.18, 0.05)	0.25	−0.06 (−0.19, 0.07)	0.36
% time spent sedentary	0.08 (−0.004, 0.153)	0.062	0.08 (0.02, 0.15)	0.014	0.16 (0.08, 0.24)	<0.001	0.21 (0.11, 0.30)	<0.001
Daily sedentary time (min)	0.005 (−0.003, 0.014)	0.23	0.01 (−0.001, 0.013)	0.09	0.01 (0.004, 0.02)	0.004	0.02 (0.01, 0.025)	0.001
% time spent on MVPA	−0.34 (−0.52, −0.17)	<0.001	−0.36 (−0.51, −0.21)	<0.001	−0.36 (−0.58, −0.15)	0.001	0.53 (−0.79, −0.28)	<0.001
Daily MVPA time (min)	−0.05 (−0.07, −0.02)	<0.001	−0.05 (−0.07, −0.03)	<0.001	−0.05 (−0–08, −0.02)	0.001	−0.07 (−0.10, −0.04)	<0.001
Maternal WC (cm)	0.51 (0.33, 0.70)	<0.001	0.12 (0.08, 0.17)	<0.001	0.11 (0.06, 0.16)	<0.001	0.14 (0.08, 0.20)	<0.001
Paternal WC (cm)	0.18 (0.12, 0.24)	<0.001	0.16 (0.11, 0.21)	<0.001	0.10 (0.04, 0.16)	<0.001	0.13 (0.06, 0.19)	<0.001

*n varies between variables due to missing data, in particular for the measures of physical activity and sedentary time, maternal WC, paternal WC.

Results derived from linear regression with correlates entered separately, adjustment for parental education was made.

MVPA: moderate to vigorous physical activity; SSB = sugar-sweetened beverages; WC = waist circumference.

#### Mediation analyses

**Figure 1:**
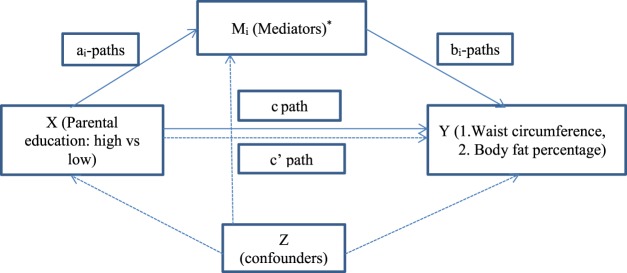
Multiple mediation model. ai paths: association between parental education (X) and the mediators (Mi), bi paths: association between the mediators (Mi) and the body weight parameter used as outcome (Y), adjusted for X, confounders (Z) and other mediators; c path: overall association between parental education (X) and outcome variable (Y), adjusted only for Z (total effect); c’ path: association between parental education X) and the outcome variable (Y) used as outcome when adjusted for Mi and Z (direct effect). *Maternal and paternal waist circumferences and TV viewing in boys; maternal and paternal waist circumferences in girls.

Figure 1 provides an outline of the hypothesized relationships between the exposure variable (parental education), potential mediators (sedentary behaviors, dietary behaviors, objectively measured PA and ST, maternal WC and paternal WC) and outcomes (adolescent WC and body fat percentage). Single mediation analyses were first conducted in which each mediator was separately entered in the analyses. In a single mediation analysis, the *a* paths represent the association between parental education and the mediator. The *b* paths represent the association between the mediator and the body weight parameter used as outcome, adjusted for parental education. The c’ path represents the association between parental education and the body weight parameter used as outcome, adjusted for the mediator. The c path represents the total association between parental education and the outcome variable. Since each mediator in the single-mediator models could be associated with omitted mediators (with unknown causal ordering) that might in turn be associated themselves with the outcome or exposure, these single-mediator models were not interpreted causally. Including the mediators jointly would obviate the need to delineate the causal ordering among these mediators under the strong assumption of no-uncontrolled-confounding (especially of the relationship between the mediator-set and the outcome in addition to those of the exposure-mediator set and the exposure-outcome relationships). Therefore, multiple mediation analyses using joint mediator models, in which all mediators were considered as a set in the effect decomposition, were conducted. In the multiple mediation analysis, the *a* paths represent the association between parental education and the mediators. The *b* paths represent the association between the mediators and the body weight parameter used as outcome (adjusted for parental education and other mediators). The *c’* path represents the association between parental education and the body weight parameter used as outcome when adjusted for the mediators. The *c* path represents the total association between parental education and the outcome variable. The following potential confounders of the exposure-mediator set, the exposure-outcome, and the mediator-set-outcome relationships were entered as covariates in the model and controlled for in all paths: family structure, ethnicity and pubertal status. However, there was no substantive impact on the results, thus unadjusted models were presented. Bias-corrected and accelerated bootstrap confidence intervals (CI) were calculated for indirect effects (a * b). Bootstrapping (5000 samples) was conducted using the PROCESS macro for SPSS by Andrew Hayes^45^. The percentage mediated was reported. Assumptions of consistency, positivity, no-uncontrolled confounding and no other sources of bias were made. It was also assumed that there was no measurement error.

Due to missing data on some variables, namely objectively measured ST and PA (1119 adolescents had valid accelerometer data), parental adiposity measures (1260 mothers and 1068 fathers participated in the study), multiple imputation procedures were used. Sensitivity analyses were conducted to compare associations obtained using multiply imputed data and those obtained using complete data.

## Results

The mean age of the participants was 11.2 years (SD = 0.3). The majority of adolescents was ethnic Norwegian and lived in two-parent families. Boys and girls with high parental education were more likely to live in two-parent families. Table 1 shows the characteristics of participants and the differences by parental education.

### Association between parental education and body weight parameters (c path)

Parental education was inversely related to adolescents’ WC and body fat percentage in both genders (Table 1).

### Association between parental education and potential correlates of body weight (a path)

Among girls, parental education was inversely related to the intake of sugar-sweetened soft drinks and unhealthy snacks. It was also inversely related to paternal and maternal WC. Among boys, parental education was inversely related to TV viewing, computer use, sugar-sweetened soft drink intake, unhealthy snack intake, maternal WC and paternal WC. There were no significant associations between the other potential mediators included and parental education (Table 1).

### Association between potential correlates and body weight parameters (b path)

Table 2 shows the results of the linear regression analyses exploring the association between potential correlates and weight parameters (adjusted for parental education).

Among girls, daily sedentary time, percent time spent sedentary, maternal WC and paternal WC were positively related to WC and body fat percentage. MVPA (daily time and percent time) was inversely related to WC and body fat percentage.

Among boys, TV viewing, computer use, maternal WC and paternal WC were positively related to WC and body fat percentage. Percent time spent sedentary was also positively related to body fat percentage. Daily MVPA time and percent time spent on MVPA were inversely related to WC and body fat percentage.

### Mediation analyses

Single mediation analyses (results not shown) found that the factors that mediated the association between parental education and both WC and body fat percentage were: paternal and maternal WC among girls, maternal WC, paternal WC and TV viewing among boys. The other factors were not found to be significant mediators in the single mediation analyses.

In the multiple mediation model among girls, maternal WC and paternal WC mediated 20% and 14% percent of the association between parental education and WC respectively. The respective proportions when body fat percentage was used as the outcome variable were 14% and 10%. Among boys, maternal WC, paternal WC and TV viewing mediated 16%, 11.5% and 13% of the association between parental education and WC. The respective proportions when body fat percentage was used as the outcome variable were 22.5%, 16% and 21% (Table 3).

**Table 3:** Multiple mediation analyses for the association between parental education and indicators of adiposity among adolescents.

	a-path	b-path	Indirect effect (a*b)	Direct effect (c’-path)	% mediated
**GIRLS**					
**Outcome: waist circumference**					
Maternal WC (cm)	−2.87 (−5.15, −0.60)	0.08 (0.03, 0.14)	−0.24 (−0.64, −0.03)		20%
Paternal WC (cm)	−2.45 (−4.52, −0.37)	0.07 (0.01, 0.13)	−0.16 (−0,48, −0.01)	−0.79 (−2.11, 0.52)	14%
Total			−0.40 (−0.84, −0.12)		33%
**Outcome: body fat %**					
Maternal WC (cm)	−2.89 (−5.16, −0.61)	0.10 (0.03, 0.17)	−0.29 (−0.76, −0.06)		14%
Paternal WC (cm)	−2.44 (−4.52, −0.36)	0.09 (0.01, 0.16)	−0.21 (−0.58, −0.02)	−1.63 (−3.18, −0.08)	10%
Total			−0.50 (−1.03, −0.16)		24%
**BOYS**					
**Outcome: waist circumference**					
Maternal WC (cm)	−3.56 (−5.85, −1,28)	0.12 (0.07, 0.18)	−0.45 (−0.93, −0.14)	−1.66 (−3.02, −0.31)	16%
Paternal WC (cm)	−2.38 (−4.46, −0.30)	0.13 (0.07, 0.20)	−0.32 (−0.80, −0.05)		11.5%
TV viewing (hrs/wk)	−2.26 (−3.72, −0.79)	0.16 (0.07, 0.24)	−0.36 (−0.82, −0.09)		13%
Total			−1.13 (−1.87, −0.59)		40.5%
**Outcome: body fat %**					
Maternal WC (cm)	−3.57 (−5.86, −1.28)	0.11 (0.06, 0.16)	−0.39 (−0.83, −0.11)	−0.71 (−1.89, 0.47)	22.5%
Paternal WC (cm)	−2.38 (−4.46, −0.30)	0.12 (0.06, 0.17)	−0.28 (−0.74, −0.05)		16%
TV viewing (hrs/wk)	−2.26 (−3.72, −0.79)	0.17 (0.09, 0.24)	−0.37 (−0.84, −0.10)		21%
Total			−1.04 (−1.76, −0.51)		59.5%

WC = waist circumference.

Only mediators significant in the single mediation models were included in the multiple mediation models.

Among girls, these mediators together mediated 33% of the association between parental education and WC; they mediated 24% of the association between parental education and body fat percentage. Among boys, the respective proportions were 40.5% and 59.5% (Table 3).

### Missing data

Sensitivity analyses comparing associations obtained using imputed data and available data showed no substantive differences.

## Discussion

In order to inform interventions aimed at reducing social inequalities in OW and OB among youth, it is vital to identify the mechanisms behind these inequalities. In the present study, parental WC was found to mediate parental educational differences in adolescent WC and body fat percentage in both genders. Parental BMI has previously been found to mediate inequalities in adiposity among youth^31^; this is in line with the findings for parental WC in the present study. These findings indicate the potential for a multigenerational “transmission” of socioeconomic differences in adiposity. The association between parental weight and child weight is itself multi-causal. It involves biological mechanisms as well as factors related to common environmental exposures, occurring in a complex gene-environment interaction context^26–28^. More studies are needed to shed light on the relative role of biological vs social/environmental factors in explaining the association between parental weight and child weight, as was also suggested in a recent review^28^. Dissociating the role of biological factors vs modifiable behavioral or environmental factors is vital for the planning as well as the timing of interventions.

TV viewing time was found to be a mediator of parental educational differences in WC and body fat percentage in boys but not in girls. Although the role of TV viewing as a mediator of socioeconomic differences in adiposity has previously been documented^31^, gender-specific differences in this regard have been less explored. TV viewing was not associated with WC and body fat percentage among girls in the present study. The association between TV viewing and body weight can be related to dietary behaviors, via mechanisms such as exposure to advertisement and passive overconsumption of foods^46^. Differences in dietary behaviors not included in this study that are related to TV viewing might thus at least partly explain the differences between genders. The findings suggest that efforts aimed at reducing screen time among boys could result in the reduction of socioeconomic differences in adiposity. Reducing screen time among youth can be a challenging task, in particular among those with low socioeconomic position (SEP). Increased screen time in this latter subgroup has been found to be related to factors such as parental time demands, concern about neighborhood safety or absence of alternative activities to TV viewing^23^. Carefully tailored interventions making use of existing knowledge about important determinants of TV viewing are thus needed.

The consumption of sugar-sweetened beverages has previously been found to mediate socioeconomic differences in adiposity^31^; this was not the case in the present study. The consumption of sugar-sweetened beverages was inversely related to parental education as also previously found in the literature^47^. However, it was not related to WC or body fat percentage. This might partly be due to differences in the type of sugar-sweetened beverages included in different studies. The reported intake of sugar-sweetened beverages in the current sample was also low. There has been an increasing awareness at the population level about the association between sugar-sweetened beverages and health. It is thus possible that there was some social desirability bias with underreporting of intake, in particular among those who were overweight/obese.

The intake of fruits and vegetables was not related to parental education; these findings are in line with those of a nationally representative survey among adolescents^48^. There was also no association between the intake of fruits and vegetables and WC as well as body fat percentage. A previous review found that there is indeterminate evidence for the mediating role of fruit and vegetable intake in the association between parental SEP and overweight/obesity^31^.

No mediating role of objectively measured PA and ST was found in this study, as these were not related to parental education. The present study contributes to the limited literature in this area which has often been based on self-reported measures of PA and SB^31^. In early adolescence, the main volume of PA is likely to be mainly non-structured/informal, as previously documented in the literature^49^. It may be that socioeconomic differences emerge later in adolescence when organized activities that might require more resources become more frequent. This hypothesis has previously also been used to explain the results of a systematic review which documented socioeconomic differences in PA levels among adolescents while such differences were not found among children (3–12 years of age)^50^. No parental educational differences in total ST were documented, although such differences were found for TV viewing and computer time. This suggests that it is the type of ST that varies by parental education and not the amount.

Higher proportions of the parental educational inequalities in adiposity were explained in boys than girls. In one of the few studies assessing gender-specific mediators of socioeconomic differences in adiposity among youth, similar findings were documented^51^. A substantial proportion of the parental educational differences in adiposity remain unexplained. This was particularly true for girls among whom only 24–33% of the association between parental education and indicators of adiposity was explained. Therefore, other mediators need to be assessed in future studies. These include factors such as a broader range of dietary behaviors, total energy intake, sleep patterns and meal patterns. Obesity is multi-causal and has multiple levels of influence; exploring the role of broader environmental factors including the availability and accessibility of opportunities for healthy lifestyle choices is warranted. Targeting such environmental factors can potentially lead to wide-reaching effects.

## Strengths and Limitations

The strengths of the study include the broad range of potential mediators included, and the objective measurement of PA, ST and anthropometric outcomes. The use of indicators of adiposity other than BMI is also a strength of the study. Gender-specific analyses were conducted, contributing to the limited evidence in this regard. Formal tests of mediation were also conducted.

However, the study should be seen in light of the following weaknesses. The cross-sectional design limits inferences about causality. However, the association between parental education and child adiposity is likely to be unilateral among youth (i.e. parental SEP affects child adiposity and not vice versa). The measurement of body fat percentage in the present study did not follow recommended procedures as the students were not in a fasting state when measurements were made. The use of self-reported measures raises validity and reliability concerns, in particular among youth. However, measures used in this study are supported by evidence of adequate test-retest reliability. Assessing dietary intake using frequency measures only can be considered to be a limitation of this study. However, the measures (except the one assessing the intake of unhealthy snacks) have previously been validated using a 7 day food record or a pre-coded food diary. Results showed that increasing frequency of intake was related to increasing amount consumed^37,38^. Self-reported measures of weight circumference can lead to underestimation; subgroup differences in this underestimation might also exist^52–54^. Such errors can affect the documented associations. Some screen-based sedentary behaviors which are increasingly prevalent among youth (e.g. use of mobile devices) were not included in the present study. Future studies should include such measures. On the other hand, measures such as the use of DVDs, which was included in this study, are unlikely to be relevant for contemporary youth.

Parental education was the only indicator of SEP included in this study. Education has been found to be the indicator of SEP most commonly related to body weight among youth^55^. It is considered to be a good measure of SEP because it might reflect both resources and knowledge affecting behaviors and is stable over time. There are associations between education and other indicators of SEP such as income and occupation in Norway^56^. Although education, income and occupation are correlated, existing research suggests that the associations between these indicators of SEP and dietary behaviors among children might be specific/independent^57^. Therefore, future studies should include different indicators of SEP.

Finally, future studies should include other potential mediators in order to better shed light on the mechanisms explaining socioeconomic differences in adiposity among youth.

## Conclusion

Parental WC was found to be a mediator of socioeconomic differences in adiposity in both genders. Identifying the underlying mechanisms behind this mediating role of parental adiposity is important from an intervention perspective. Dissecting the possible role of genetic factors as well as shared social and environmental factors in the association between parental weight and child weight is vital in this regard. Furthermore, reducing time spent watching TV, in particular among those with low parental education, could contribute to reducing social inequalities in adiposity among boys. The study, using objective measures, also found that PA and sedentary time do not play a role in explaining socioeconomic differences in adiposity in the study setting. A significant percentage of the association between parental education and adiposity among the adolescents remained unexplained. Thus, future studies including other potential mediators are warranted.

## Data Availability

The datasets analysed during the current study are available from the corresponding author on reasonable request.
